# Supplementary figures and images for: Species-specific emergence of H7 highly pathogenic avian influenza virus is driven by intrahost selection differences between chickens and ducks
Source: PLoS Pathog. 2024 Feb 26;20(2):e1011942. doi: 10.1371/journal.ppat.1011942 (PMC10919841; doi:10.1371/journal.ppat.1011942)

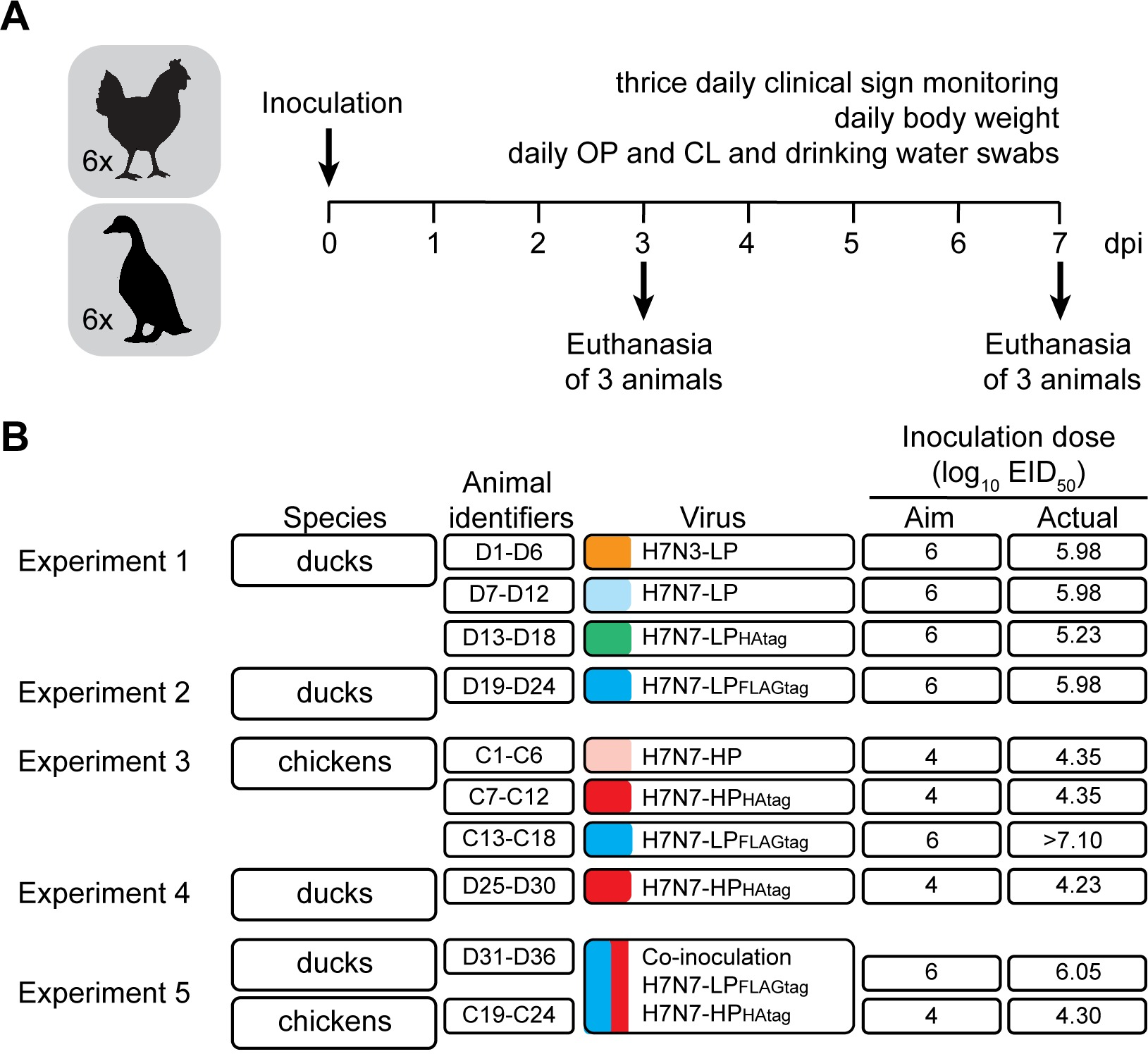

Supplement: S1 Fig — (A) Schematic overview of the experimental design of the in vivo experiments. Six six-week-old chickens or ducks were inoculated intratracheally and intra-oesophageally with H7 avian influenza viruses. Disease severity was monitored thrice daily and body weight measurements, oropharyngeal (OP), cloacal (CL), and drinking water swabs were collected daily. Three animals were euthanized at three dpi and at seven dpi, or at earlier time points when animals reached humane endpoints, to harvest tissues for virological and histological analysis. (B) Overview of chicken and duck mono- and co-inoculation groups as divided over five experiments, indicating species, animal identifiers, virus, intended inoculation dose (aim), and actual inoculation dose as determined by endpoint titration of the inoculum in embryonated chicken eggs and expressed as log10 EID50. (TIF) [file ppat.1011942.s001.tif]

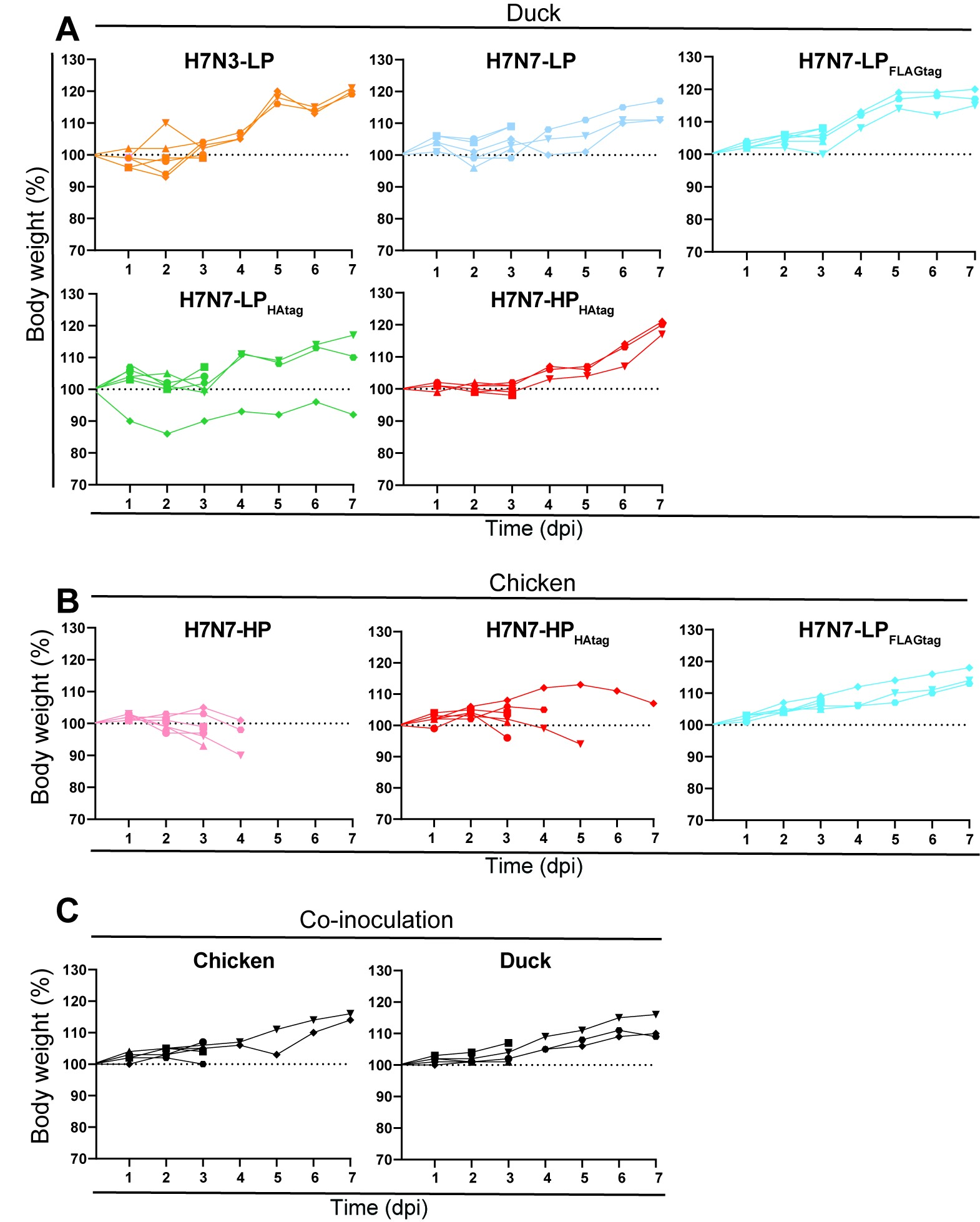

Supplement: S2 Fig — Body weight was recorded daily in mono-inoculated ducks (A) and chickens (B) and co-inoculated chickens and ducks (C) and is presented relative to the body weight on the day of inoculation. (TIF) [file ppat.1011942.s002.tif]

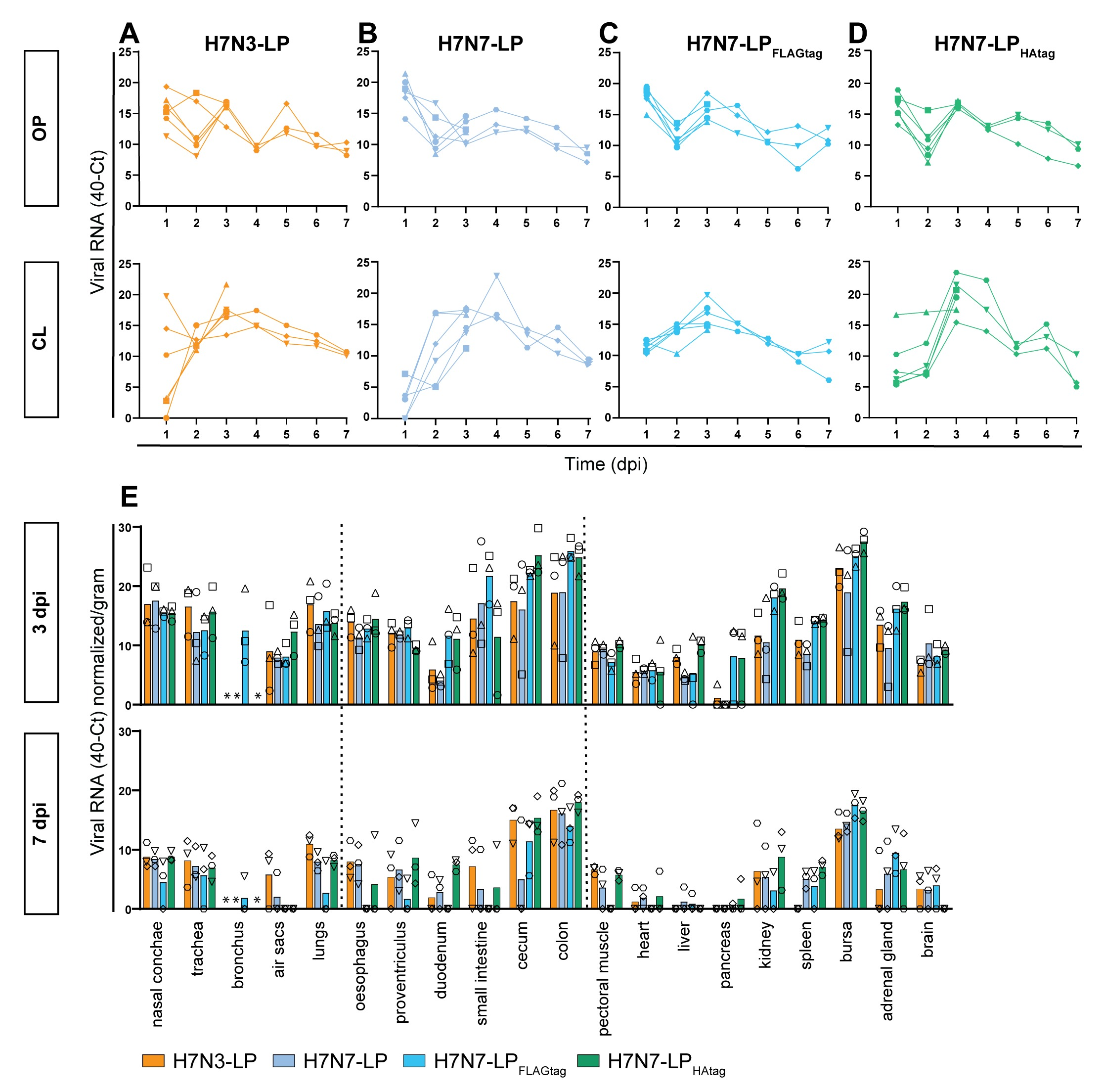

Supplement: S3 Fig — (A-D) Viral RNA quantification in oropharyngeal (OP (top panels)) and cloacal (CL (bottom panels)) swabs of ducks mono-inoculated with ~106 EID50 of H7N3-LP (A), H7N7-LP (B), H7N7-LPFLAGtag (C), or H7N7-LPHAtag (D). Viral RNA amounts were determined by RT-qPCR targeting the influenza virus matrix segment and expressed as 40-cycle threshold (Ct). (E) Viral RNA quantification in tissues harvested at 3 and 7 dpi from ducks inoculated with ~106 EID50 of H7N3-LP, H7N7-LP, H7N7-LPFLAGtag, or H7N7-LPHAtag. Viral RNA amounts were determined by RT-qPCR targeting the influenza virus matrix segment and expressed as 40-Ct normalized/gram tissue. Bars represent the arithmetic mean and symbols indicate data from individual animals as in (A-D). The dotted lines distinguish tissues from the respiratory, digestive, and miscellaneous systems. The asterisks indicate that data are absent. (TIF) [file ppat.1011942.s003.tif]

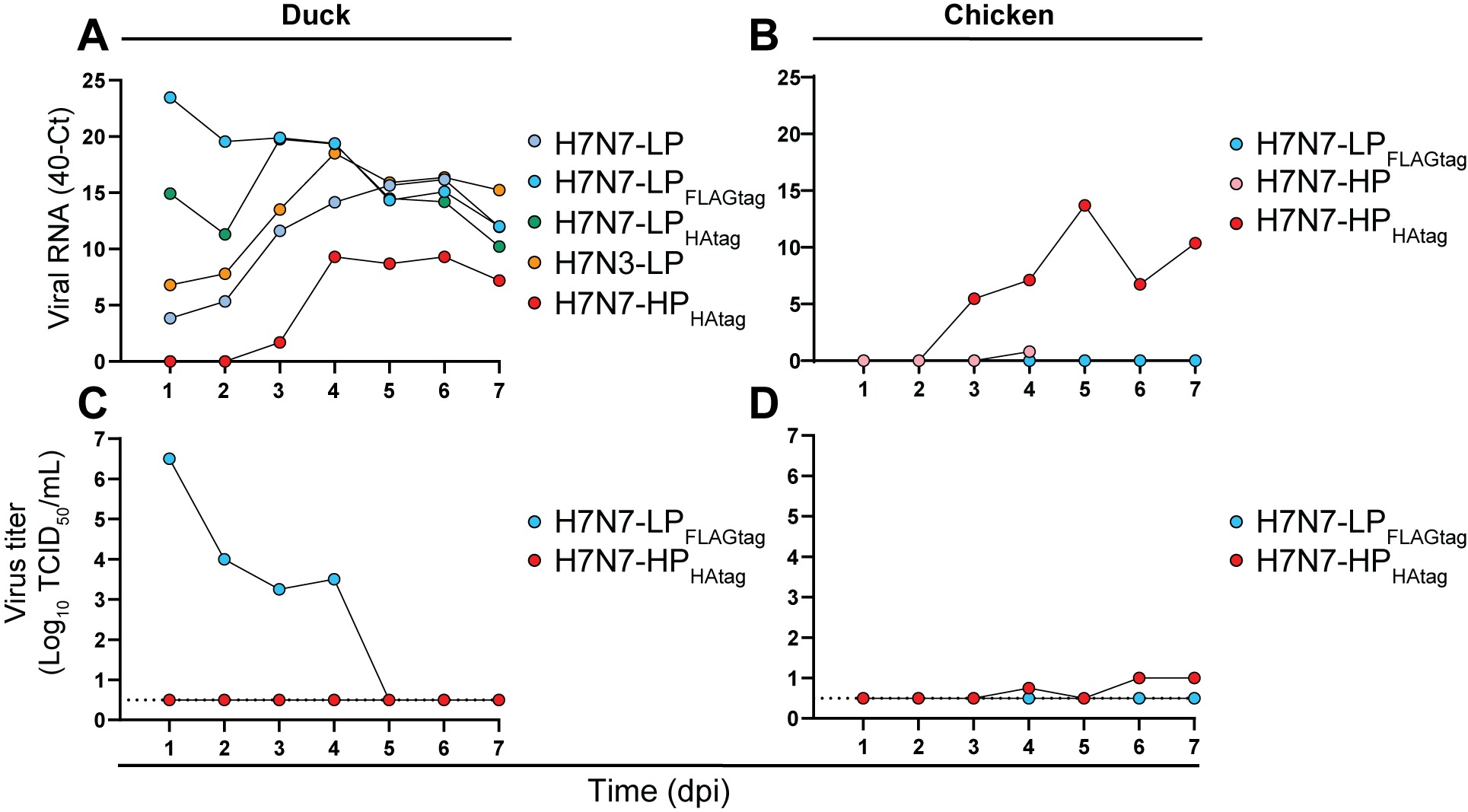

Supplement: S4 Fig — (A) Viral RNA quantification in swabs taken from the drinking water of ducks mono-inoculated with ~106 EID50 of H7N7-LP, H7N7-LPFLAGtag, H7N7-LPHAtag, or H7N3-LP, or ~104 EID50 of H7N7-HPHAtag. Viral RNA amounts were determined by RT-qPCR targeting the influenza virus matrix segment and expressed as 40-cycle threshold (Ct). (B) Viral RNA quantification in swabs taken from the drinking water of chickens mono-inoculated with ~104 EID50 of H7N7-HP or H7N7-HPHAtag, or ~106 EID50 of H7N7-LPFLAGtag, and analyzed as in (A). (C, D) Infectious virus titers in swabs taken from the drinking water of ducks (C) and chickens (D) mono-inoculated with ~104 EID50 of H7N7-HPHAtag or ~106 EID50 of H7N7-LPFLAGtag. Infectious titers were determined by endpoint titration in MDCK cells and expressed as log10 TCID50/mL. The horizontal dotted lines indicate the limit of detection of the endpoint titration assay. (TIF) [file ppat.1011942.s004.tif]

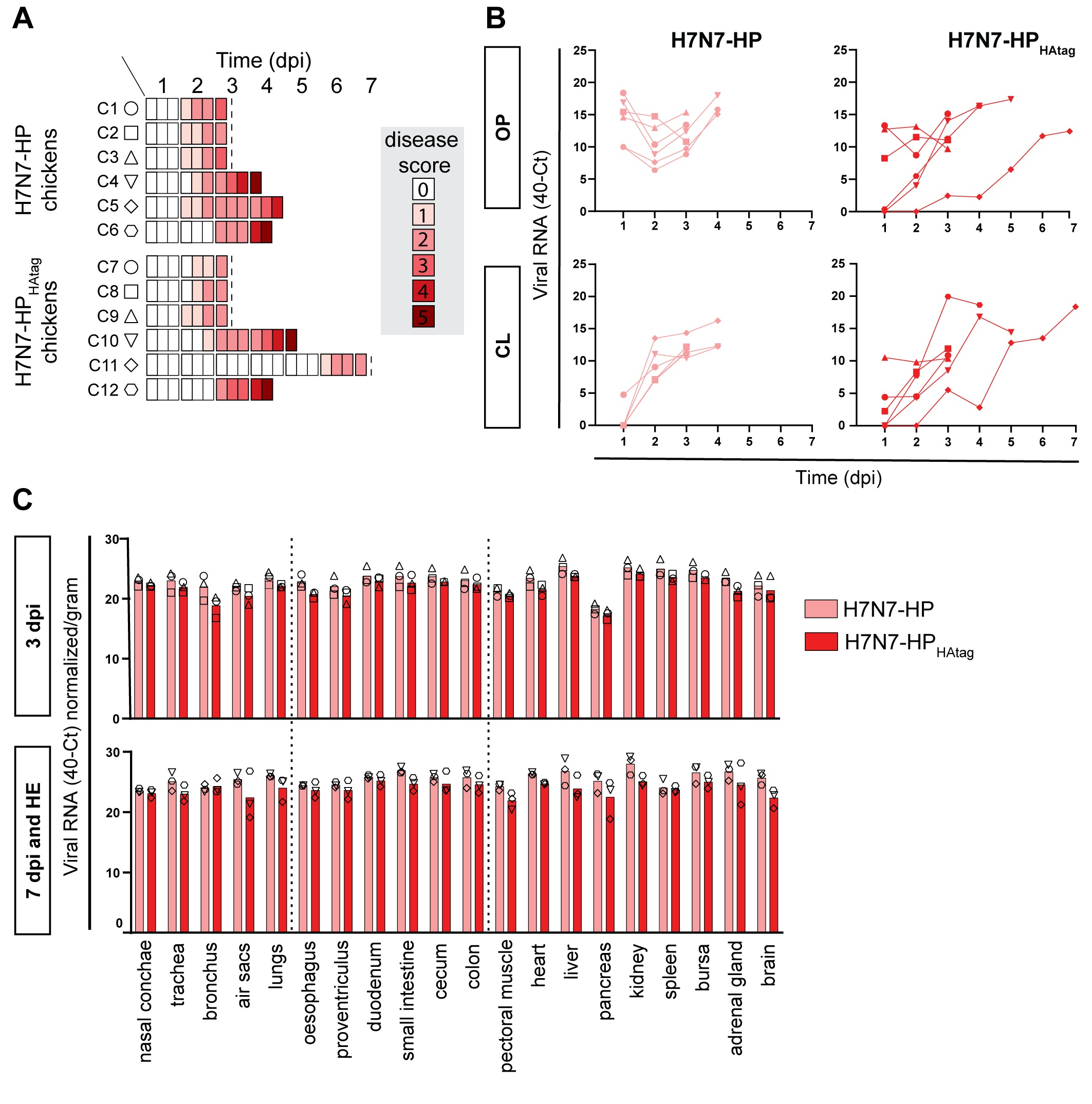

Supplement: S5 Fig — (A) Severity of disease in six chickens inoculated with ~104 EID50 of H7N7-HP (C1-C6) or H7N7-HPHAtag (C7-C12). Disease severity scores (0–5) were determined thrice daily, each box representing one time point. Dotted lines indicate planned time points for euthanasia and dissection. (B) Viral RNA quantification in oropharyngeal (OP) and cloacal (CL) swabs of individual chickens mono-inoculated with H7N7-HP or H7N7-HPHAtag. Viral RNA amounts were determined by RT-qPCR targeting the influenza virus matrix segment and expressed as 40-cycle threshold (Ct). Symbols are as in (A). (C) Viral RNA quantification in tissues harvested at 3 dpi, humane endpoint (HE; C4, C5, C6, C10, and C12), or 7 dpi (C11) from chickens inoculated with ~104 EID50 of H7N7-HP or H7N7-HPHAtag. Viral RNA amounts were determined by RT-qPCR targeting the influenza virus matrix segment and expressed as 40-Ct normalized/gram tissue. Bars represent the arithmetic mean and symbols indicate data from individual animals as in (A, B). The dotted lines distinguish tissues from the respiratory, digestive, and miscellaneous systems. (TIF) [file ppat.1011942.s005.tif]

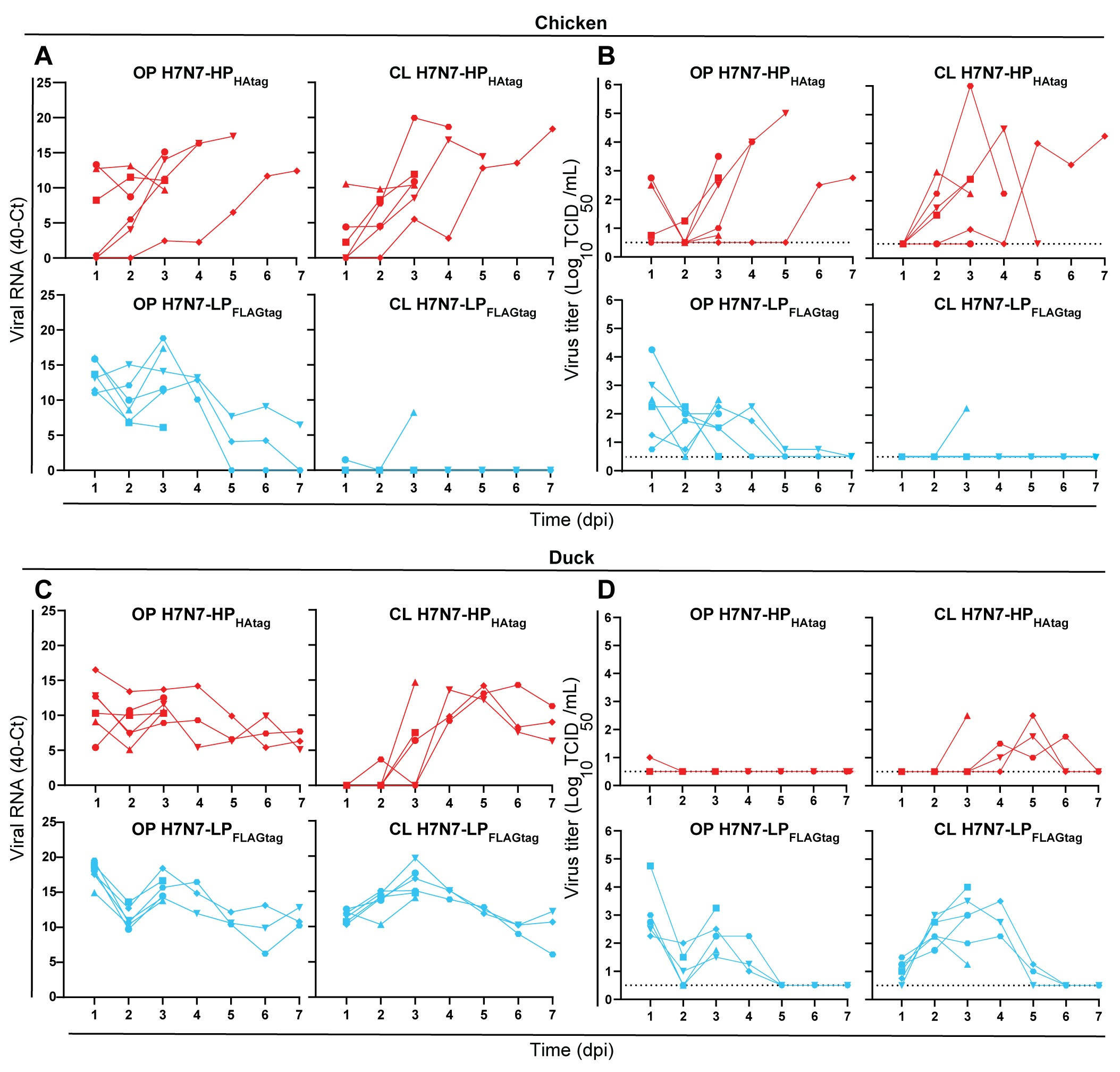

Supplement: S6 Fig — (A, C) Viral RNA quantification in oropharyngeal (OP) and cloacal (CL) swabs of chickens (A) and ducks (C) mono-inoculated with ~104 EID50 of H7N7-HPHAtag or ~106 EID50 of H7N7-LPFLAGtag. Viral RNA amounts were determined by RT-qPCR targeting the influenza virus matrix segment and expressed as 40-cycle threshold (Ct). The symbols indicate data from an individual animal. (B, D) Infectious virus titers in the swabs from (A, C). Infectious titers were determined by endpoint titration in MDCK cells and expressed as log10 TCID50/mL. Symbols are as in (A, C). The horizontal dotted lines indicate the limit of detection of the endpoint titration assay. (TIF) [file ppat.1011942.s006.tif]

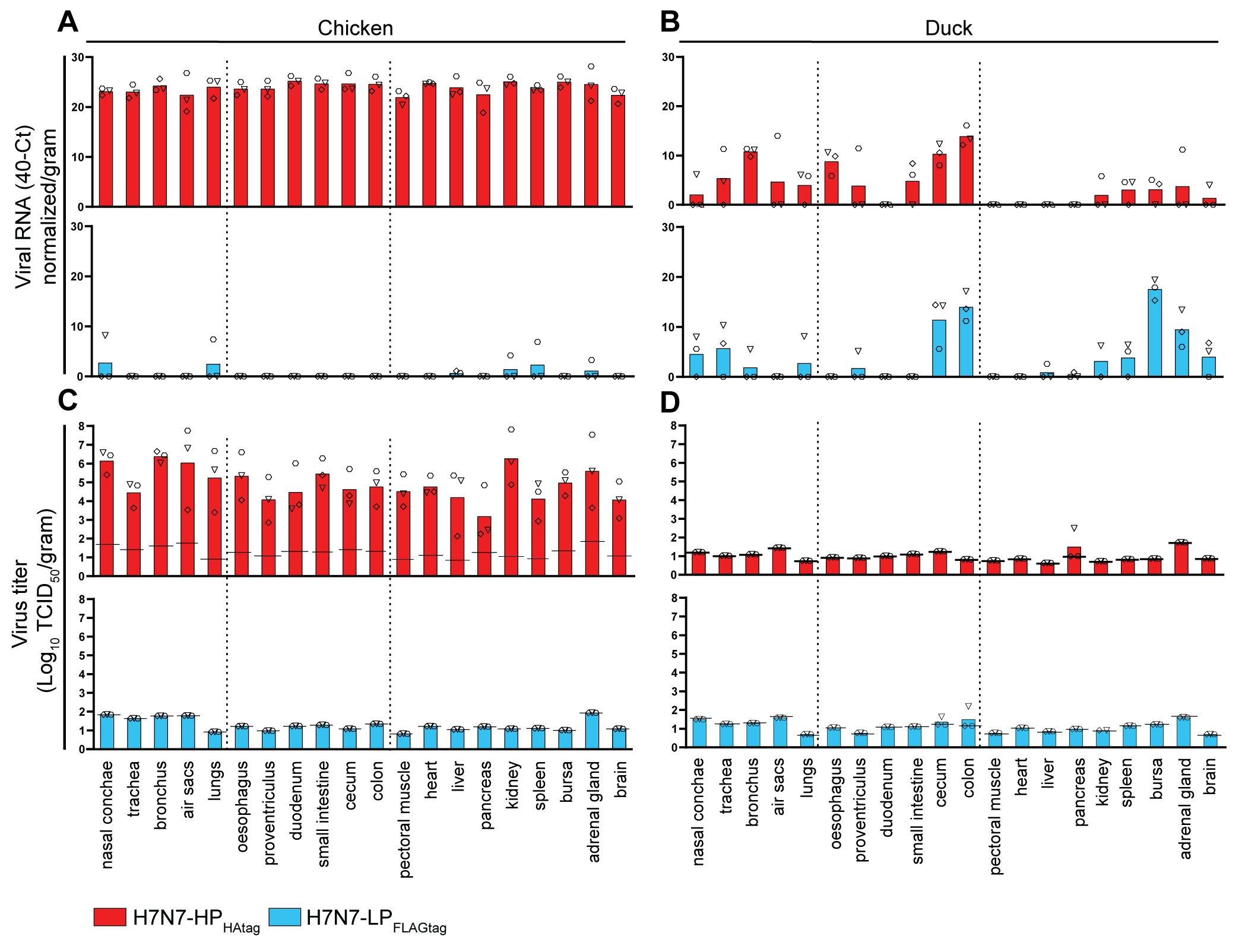

Supplement: S7 Fig — (A, B) Viral RNA quantification in tissues harvested at 7 dpi or at humane endpoint from chickens (A) and ducks (B) inoculated with ~106 EID50 H7N7-LPFLAGtag or ~104 EID50 H7N7-HPHAtag. Viral RNA amounts were determined by RT-qPCR targeting the influenza virus matrix segment and expressed as 40-cycle threshold (Ct) normalized/gram tissue. Bars represent the arithmetic mean and symbols indicate data from individual animals. The dotted lines distinguish tissues from the respiratory, digestive, and miscellaneous systems. (C, D) Infectious virus titers in the tissues from (A, B). Infectious titers were determined by endpoint titration in MDCK cells and expressed as log10 TCID50/gram tissue. Bars represent the arithmetic mean of log10 transformed values. Symbols and dotted lines similar to (A, B). The horizontal solid lines indicate the limit of detection of the endpoint titration assay per tissue. (TIF) [file ppat.1011942.s007.tif]

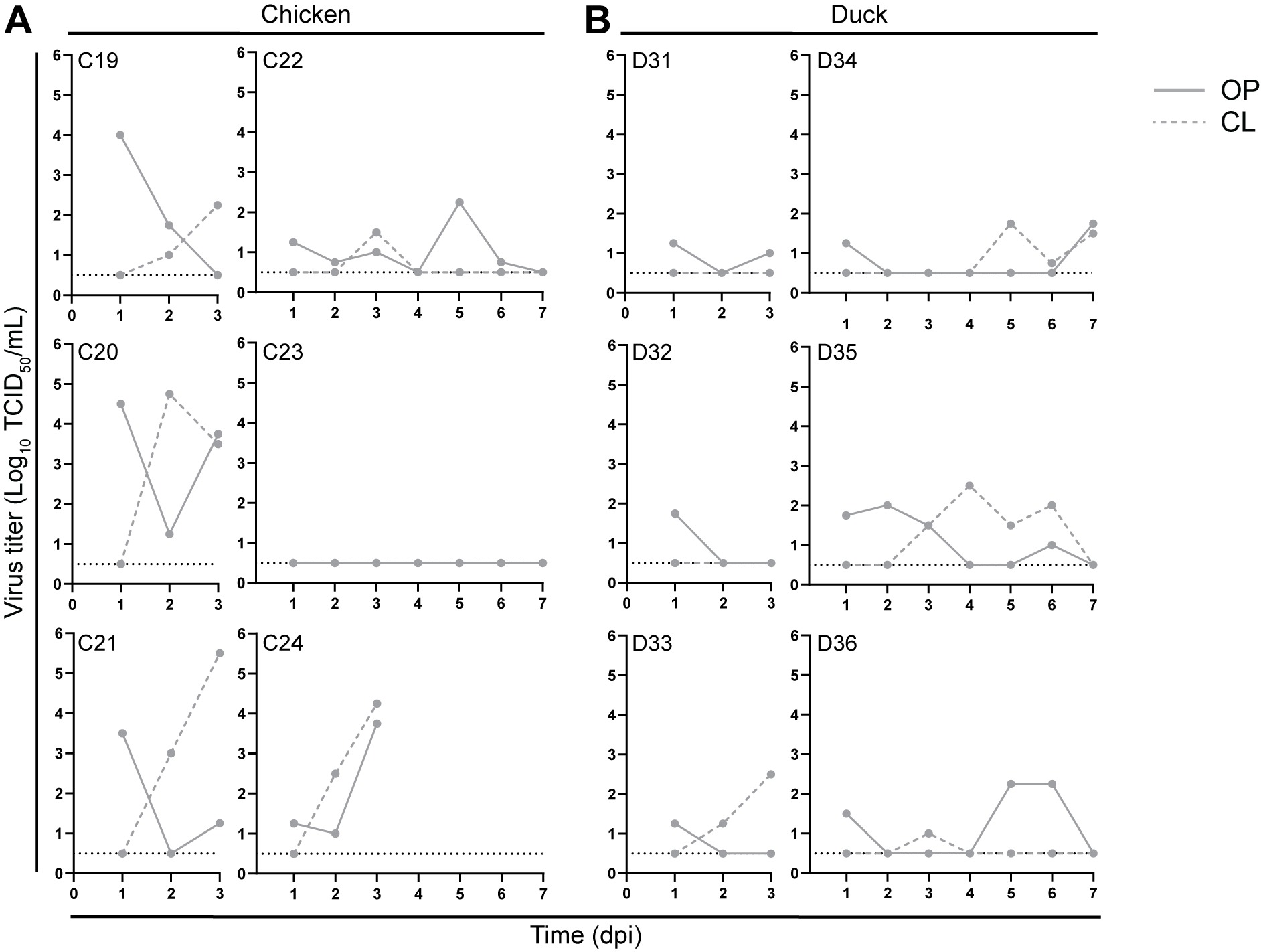

Supplement: S8 Fig — Infectious virus quantification in oropharyngeal (OP; solid lines) and cloacal (CL; dotted lines) swabs from H7N7-LPFLAGtag/H7N7-HPHAtag co-inoculated chickens (A) and ducks (B). Infectious titers were determined by endpoint titration in MDCK cells and expressed as log10 TCID50/mL (viral RNA levels and plaque forming units are depicted in Figs 4 and 5). (TIF) [file ppat.1011942.s008.tif]

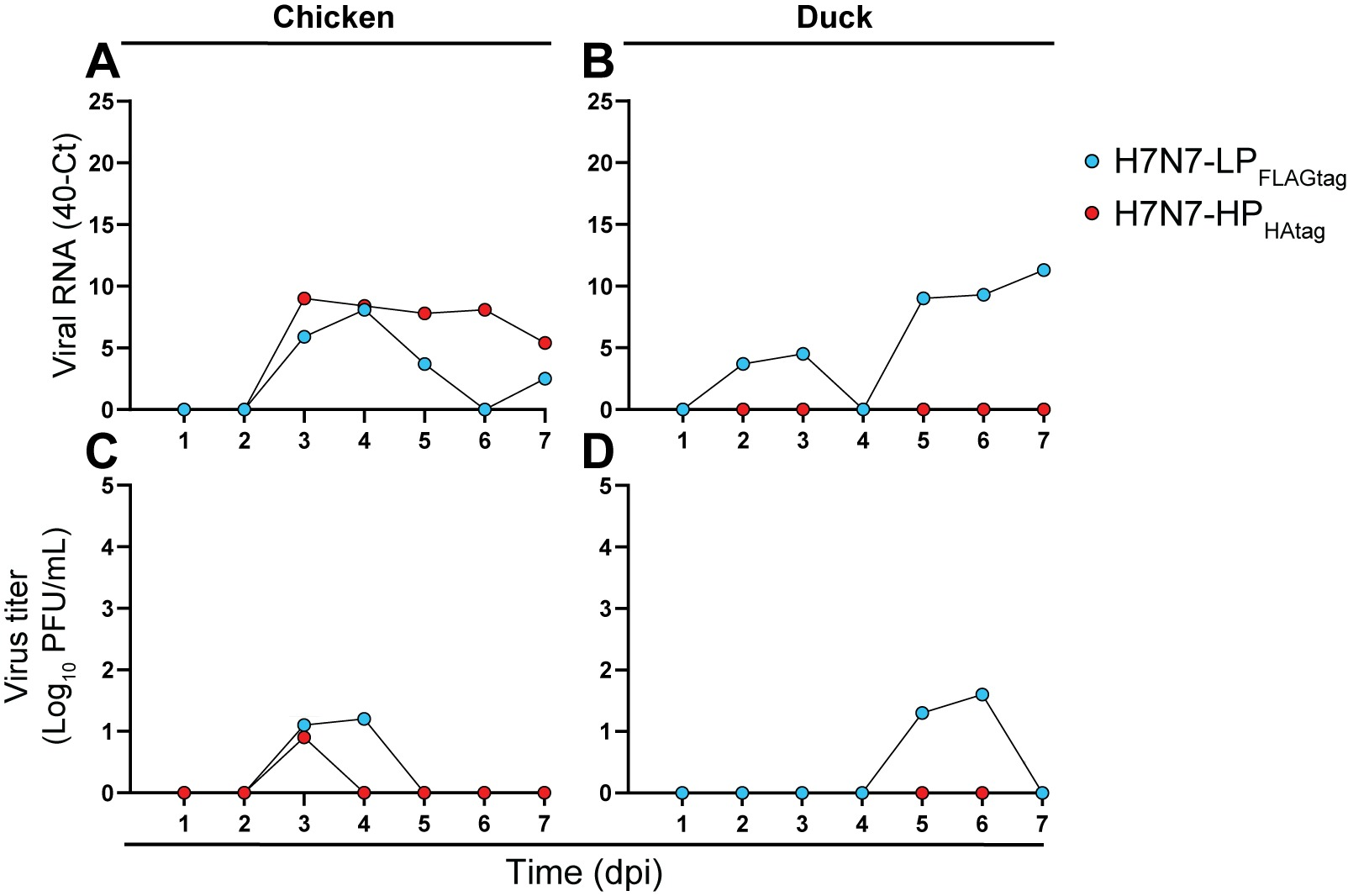

Supplement: S9 Fig — Viral RNA quantification in swabs taken from the drinking water of H7N7-LPFLAGtag/H7N7-HPHAtag co-inoculated chickens (A) and ducks (B). Viral RNA amounts were determined by HPAIV/LPAIV differentiating RT-qPCR targeting the HA cleavage site and expressed as 40-Ct. (C, D) Infectious virus titers in the swabs from (A, B), depicted as log10 PFU/mL as measured by the HPAIV/LPAIV differentiating plaque assay. (TIF) [file ppat.1011942.s009.tif]

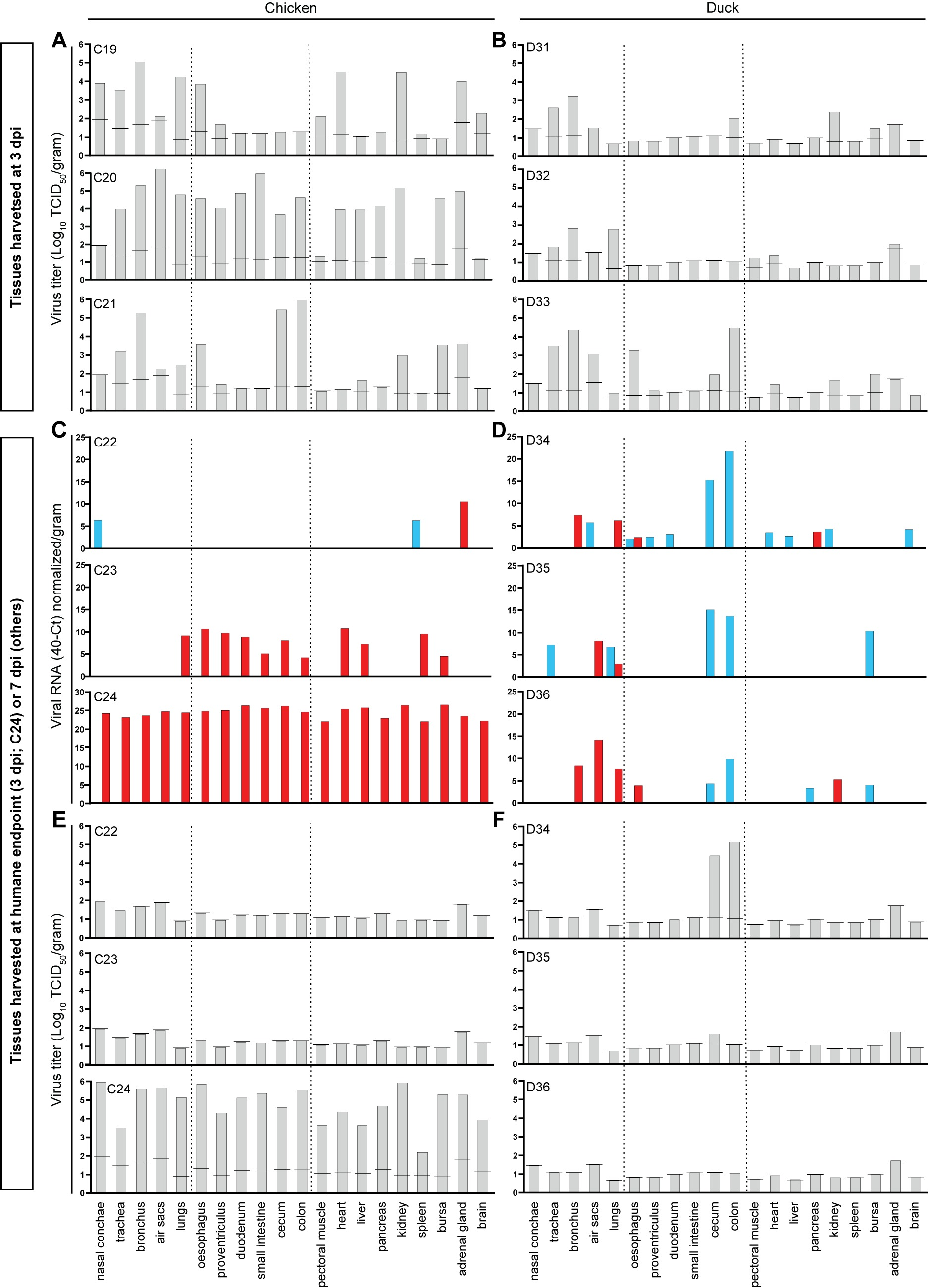

Supplement: S10 Fig — (A, B) Infectious virus titers in tissues harvested at 3 dpi from chickens (A) and ducks (B) co-inoculated with H7N7-HPHAtag and H7N7-LPFLAGtag. Infectious titers were determined by endpoint titration in MDCK cells and expressed as log10 TCID50/gram tissue. The horizontal solid lines indicate the limit of detection of the endpoint titration assay per tissue. The dotted lines distinguish tissues from the respiratory, digestive, and miscellaneous systems. (C, D) Viral RNA quantification in tissues harvested at humane endpoint or at 7 dpi from chickens (C) and ducks (D) co-inoculated with H7N7-HPHAtag and H7N7-LPFLAGtag. Viral RNA amounts were determined by HPAIV/LPAIV differentiating RT-qPCR targeting the HA cleavage site region and expressed as 40-cycle threshold normalized per gram tissue. Dotted lines are similar as in (A, B). (E, F) Infectious virus titers in the tissues from (C, D). Infectious titers were determined as in (A, B). Dotted and horizontal solid lines are similar as in (A, B). (TIF) [file ppat.1011942.s010.tif]

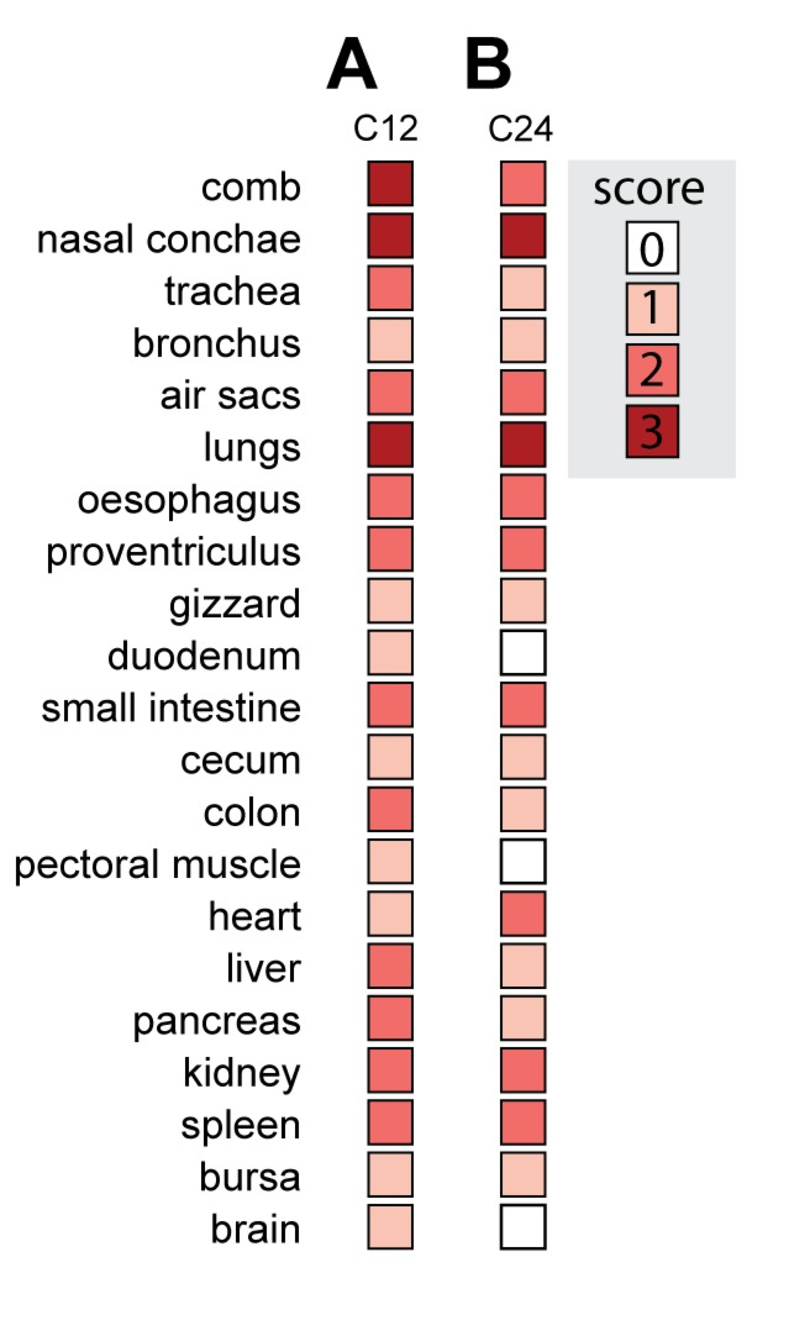

Supplement: S11 Fig — (A) Degree of viral antigen expression in tissues of a chicken mono-inoculated with ~104 EID50 H7N7-HPHAtag and euthanized at humane endpoint at 4 dpi (A) and a chicken co-inoculated with H7N7-LPFLAGtag/H7N7-HPHAtag and euthanized at humane endpoint at 3 dpi (B). Viral antigen expression was determined by immunohistochemical detection of the influenza nucleoprotein. Tissues showing no positivity were scored as (0), those showing sporadic single positive cells as (1), multiple sites of positive cells as (2), and diffuse positivity as (3). (TIF) [file ppat.1011942.s011.tif]

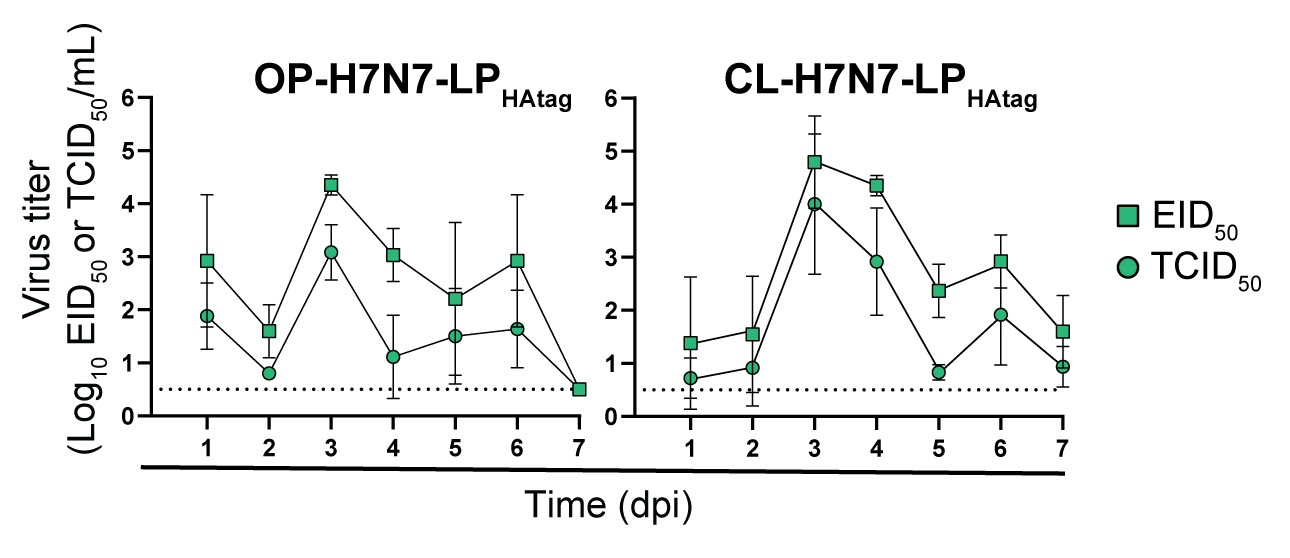

Supplement: S12 Fig — Infectious virus titers in oropharyngeal (OP) and cloacal (CL) swabs, as determined by endpoint titration assay in embryonated chicken eggs or in MDCK cells, of the three ducks inoculated with ~106 EID50 H7N7-LPHAtag that were followed for seven days (D16-D18). Titers are expressed as log10 EID50/mL or as log10 TCID50/mL. Data are depicted as arithmetic mean ± SD calculated from log10 transformed values. The horizontal dotted lines indicate the limit of detection of the endpoint titration assays. (TIF) [file ppat.1011942.s012.tif]
