# Supplementary material for: Species-specific emergence of H7 highly pathogenic avian influenza virus is driven by intrahost selection differences between chickens and ducks
Source: PLoS Pathog. 2024 Feb 26;20(2):e1011942. doi: 10.1371/journal.ppat.1011942 (PMC10919841; doi:10.1371/journal.ppat.1011942)
Supplement: S1 Table — (DOCX) [file ppat.1011942.s013.docx]

**S1 Table. Assessment of virus replication outside of the respiratory and digestive tracts of ducks.**

| *Species* | *Virus* | *Animal number* | *Tissue* | *Viral RNA (40-Ct adjusted/ gram)*  *Matrix* | *Viral RNA (40-Ct adjusted/ gram)*  *HPAIV/LPAIV* | *Infectious virus*  *(log_10_ TCID_50_/ gram)* | *Infectious virus*  *(log_10_ PFU/gram) HPAIV/LPAIV* | *IHC^a^* | *RNA ISH^b^* |
| --- | --- | --- | --- | --- | --- | --- | --- | --- | --- |
| Ferret | pH1N1 | N.A.^c^ | Lungs | N.D.^d^ | N.A. | N.D. | N.A. | +++ | +++ |
| Chicken | H7N7-LP_FLAGtag_/ H7N7-HP_HAtag_ | C24 | Comb | N.D. | N.D. | N.D. | N.D. | +++ | +++ |
| Duck | H7N7-LP_FLAGtag_ | D19 | Kidney | 19.9 | N.D. | 4.56 | N.D. | - | - |
| Duck | H7N7-LP_FLAGtag_ | D20 | Kidney | 18.6 | N.D. | 4.67 | N.D. | - | - |
| Duck | H7N7-HP_HAtag_ | D25 | Kidney | 15.5 | N.D. | 1.36 | N.D. | - | - |
| Duck | H7N7-HP_HAtag_ | D26 | Air sacs | 25.0 | N.D. | 4.45 | N.D. | + | ++ |
|  |  |  | Kidney | 15.8 | N.D. | 1.51 | N.D. | - | - |
|  |  |  | Heart | 16.0 | N.D. | 2.72 | N.D. | - | - |
| Duck | H7N7-LP_FLAGtag_/ H7N7-HP_HAtag_ | D31 | Kidney | 16.3 | 15.6/- | 2.38 | 2.3/- | - | - |
| Duck | H7N7-LP_FLAGtag_/ H7N7-HP_HAtag_ | D32 | Pectoral muscle | 14.9 | 13.8/- | 1.24 | -/- | - | - |
|  |  |  | Heart | 9.6 | 9.3/- | 1.37 | -/- | - | - |
|  |  |  | Kidney | 14.2 | 13.3/- | <0.82^e^ | -/- | - | - |
| Duck | H7N7-LP_FLAGtag_/ H7N7-HP_HAtag_ | D33 | Heart | 11.5 | 11.1/- | 1.45 | -/- | - | - |
|  |  |  | Kidney | 13.6 | 13.6/- | 1.68 | 1.3/- | - | - |

^a^ IHC = immunohistochemistry detecting influenza virus nucleoprotein. Tissues showing no positivity were scored as (-), those showing sporadic single positive cells as (+), multiple sites of positive cells as (++), and diffuse positivity as (+++), in accordance with Fig. 6.

^b^ RNA ISH = RNA in-situ hybridization assay detecting influenza A virus matrix and nucleoprotein vRNA was performed and tissue positivity was scored as in footnote (^a^).

^c^ N.A. = not applicable.

^d^ N.D. = not determined.

^e^ Below the limit of detection (10^0.82^ TCID_50_/gram).
