# Supplementary material for: Species-specific emergence of H7 highly pathogenic avian influenza virus is driven by intrahost selection differences between chickens and ducks
Source: PLoS Pathog. 2024 Feb 26;20(2):e1011942. doi: 10.1371/journal.ppat.1011942 (PMC10919841; doi:10.1371/journal.ppat.1011942)
Supplement: Attachment — Submitted filename: de Bruin review.docx [file ppat.1011942.s014.docx]

**Disclosure**: I am not an expert on avian influenza. I do have expertise on mammalian influenza viruses.

In this manuscript, de Bruin et al. posed a clear and testable hypothesis: that intrahost selection of HPAIVs is favoured in poultry and not in ducks. They also hypothesize that this is due to differences between bird species in virus tropism, ability to disseminate systemically and virus replication. To test their hypothesis, the authors performed a pathogenesis study to examine the process of selection of highly pathogenic avian influenza virus (HPAIV) in vivo, using a compare/contrast approach based on experimental infections and coinfections of chicken and ducks. Specifically, they used two isogenic viruses generated by reverse genetics: the wild-type H7N7 HPAIV virus (A/chicken/Netherlands/1/03) and a low pathogenic version in which the HA polybasic region of the virus was replaced by a low-pathogenic sequence. The viruses used were further modified by the addition of epitope tags to allow discrimination between them.

Chickens and ducks were subject to experimental inoculations with HPAIV and LPAIV viruses in single and coinfections. Inoculated animals were monitored for clinical signs; virus shedding was measured by qPCR and titration of infectious virus; and distribution of virus antigens was examined the respiratory and the gastrointestinal tract, as well as other tissues.

The study is very well designed and executed. The in vivo nature of the study, using chickens and ducks, which are natural hosts of avian influenza viruses, gives this study a very high biological relevance. I think that, overall, the results support their hypothesis. The interindividual variation observed in shedding and virus detection in tissues is expected, so I do not have a problem with it.

I found the manuscript difficult to follow, but this might be simply because there is so much data presented. However, some points could have been clearer. For example, the sentence that describes the process of HPAIV emergence (genesis, selection, and transmission, line 482) should be in the introduction as it would help setting up the focus of the study.

My main criticism about the study is that the authors did not go a little bit deeper to identify the main processes involved in differential selection of HPAIV and LPAIV in chicken and ducks. Since they have RNA from tissues, as well as FFPE sections, they could have explored differences in the host response to infection and coinfection that might explain the observed results. For example, are there differences in the IFN response in tissues? Are there differences in inflammatory responses and/or lesions that could explain the observed differences in virus antigen distribution? Transcriptomics or immunostaining approaches could provide important information.

Another main comment is about novelty. The authors mention in their discussion that their results are mainly consistent with previous studies. They also mention some differences with other studies but discuss potential differences appropriately. As I am not an avian influenza expert, I cannot say if the results presented are of enough novelty for PLOS PATH.

Minor comments:

- It is a shame that the FLAG-tag signal was non-specific. As a result, the identification of the FLAG-tagged virus was inferred rather than directly detected.

- It would be good to state it in the paper the rationale for the 1:62 ratio of HP/LP viruses in coinfections.

- What was the criterion for tissue sample collection? Did you collect tissues from areas that exhibited macroscopic lesions? This is important for organs such as the intestines that are quite large.

- Regarding qPCRs, was viral genomic RNA quantified? If yes, it would be simpler to present the results as genome copies/gram of tissue. I don't know how to interpret the RNA quantities as presented --> (40-Ct) normalized/gram.

-Have the viruses been sequenced when collected from the animals? Were their sequences the same as what was inoculated? Is it possible that the interindividual variations observed in virus shedding and virus distribution were due to changes in the virus at the consensus level?

- The graphs should have the same scale in the Y axis for all the figures.

- In figure 6, I do not understand the difference between a score of 0 and "not present". Please clarify.

Editorial changes and clarifications suggested:

-Replace sacrificed with euthanised throughout the text of the paper (sacrifice has religious implications).

-For each result it would be good include a final sentence summarising the overall result. This was done for some results but not for others. Given the complexity of the experiments and data presented I think this is very useful even if it may sound repetitive.

- Line 375: "In co-inoculated ducks, NP expression was detected in a few cells in the air sacs and colon (Fig 6B), which constituted of too few cells to robustly perform the HAtag immunostaining." However, in Figure S12 you show a single cell that is positive for NP and on the next serial section you show a single cell that is positive for HAtag. There seems to be no reason not do the HAtag staining.

- Line 436: "Here, the replication of H7N7-HPHAtag in air sac epithelial cells of co-inoculated chickens at 3 dpi was striking. " Please explain why it was striking.

Line 456: "in tissues outside the respiratory and digestive tracts in ducks suggesting". Is this referring to coinfected ducks? Or monoinfected? Please clarify in the text.

Line 460: "Here, we have found no convincing evidence of active LPAIV or HPAIV replication,

either by detection of viral antigen or genomic viral RNA, outside of the respiratory and digestive tracts of ducks." I think Figures 3 and 5 contradict this statement. Figure 3 shows RNA of both HP and LP viruses in adrenal gland, bursa, and kidney. If I am wrong, please clarify what is meant by "convincing" evidence.
