# Supplementary material for: Species-specific emergence of H7 highly pathogenic avian influenza virus is driven by intrahost selection differences between chickens and ducks
Source: PLoS Pathog. 2024 Feb 26;20(2):e1011942. doi: 10.1371/journal.ppat.1011942 (PMC10919841; doi:10.1371/journal.ppat.1011942)
Supplement: Attachment — Submitted filename: Response to Reviewers PLOS pathogens.docx [file ppat.1011942.s015.docx]

Dear Dr. Richard,

Thank you very much for submitting your manuscript "Species-specific emergence of highly pathogenic avian influenza virus is driven by intrahost selection differences between chickens and ducks" for consideration at PLOS Pathogens. As with all papers reviewed by the journal, your manuscript was reviewed by members of the editorial board and by several independent reviewers. In light of the reviews (below this email), we would like to invite the resubmission of a significantly-revised version that takes into account the reviewers' comments.

While all reviewers thought this study may be of interest to the broader community, they also suggested additional revisions that would improve the quality of the ms. We concur. We also believe that those revisions may require additional experimentation due to concerns about the low number of chickens and ducks used in the experiments, and significant variation in their experimental setup. Additional experiments would strengthen the findings.

We cannot make any decision about publication until we have seen the revised manuscript and your response to the reviewers' comments. Your revised manuscript is also likely to be sent to reviewers for further evaluation.

When you are ready to resubmit, please upload the following:

[1] A letter containing a detailed list of your responses to the review comments and a description of the changes you have made in the manuscript. Please note while forming your response, if your article is accepted, you may have the opportunity to make the peer review history publicly available. The record will include editor decision letters (with reviews) and your responses to reviewer comments. If eligible, we will contact you to opt in or out.

[2] Two versions of the revised manuscript: one with either highlights or tracked changes denoting where the text has been changed; the other a clean version (uploaded as the manuscript file).

Important additional instructions are given below your reviewer comments.

Please prepare and submit your revised manuscript within 60 days. If you anticipate any delay, please let us know the expected resubmission date by replying to this email. Please note that revised manuscripts received after the 60-day due date may require evaluation and peer review similar to newly submitted manuscripts.

Thank you again for your submission. We hope that our editorial process has been constructive so far, and we welcome your feedback at any time. Please don't hesitate to contact us if you have any questions or comments.

Sincerely,

Daniel R. Perez, PhD
Academic Editor
PLOS Pathogens

Benhur Lee
Section Editor
PLOS Pathogens

Kasturi Haldar
Editor-in-Chief
PLOS Pathogens
orcid.org/0000-0001-5065-158X

Michael Malim
Editor-in-Chief
PLOS Pathogens
orcid.org/0000-0002-7699-2064

***********************

Dear Dr. Richard et al,

I have read the comments of the reviewers and read the ms myself. The reviewers have recommended "Major Revisions" that I believe will improve the quality of the ms. I also believe that those revisions may require additional experimentation due to concerns about the low number of chickens and ducks used in the experiments, and significant variation in their experimental setup. I think additional experiments would strengthen the findings.

We thank the editor for expressing his opinion that additional animal experimentation would only strengthen our findings, but may not be essential. We have indicated for each review point why we are of the opinion that repeating the here-described animal experiments would not substantially strengthen the manuscript in such a manner that it would justify additional animal experimentation. We hope that, considering our responses to the reviewers’ concerns, we have convinced the editor that the revised manuscript is acceptable for publication in PLOS Pathogens.

**Response to Reviewers**

We thank the reviewers for their constructive critiques. Please find their comments reproduced below, followed up by each of our answers in a green font. The line numbers mentioned in each of the answers refer to the line numbers within the file “Marked-up manuscript”.

**Part I – Summary**

*Please use this section to discuss strengths/weaknesses of study, novelty/significance, general execution and scholarship.*

**Reviewer #1:** Low pathogenic avian influenza viruses (LPAIV) of the H5 and H7 subtypes can emerge as HPAIV through the acquisition of a multibasic cleavage site in hemagglutinin. Interestingly, HPAIV have been shown to only emerge in poultry species but not in wild aquatic birds. In this manuscript by de Bruin et al., the authors propose and test the hypothesis that the emergence of HPAIV in poultry is due to species specific selection advantages and that the absence of HPAIV emergence in wild birds is due to selection disadvantages. The authors test this hypothesis by co-inoculating differentially tagged isogenic HPAIV and LPAIV strains at a 1:62 ratio into chickens and ducks, and assess the levels of HPAIV and LPAIV at different times post infection. In four out of six co-inoculated chickens, HPAIV dominated the infection. In contrast, predominantly LPAIV was detected in co-inoculated ducks. Based on these results, the authors conclude that the selection of HPAIV is favored over LPAIV in chickens whereas HPAIV is disfavored in ducks. Overall, this is a very detailed study that provides insight into the emergence of HPAIV only in poultry but not in wild aquatic birds. The authors should address the following concerns.

**Reviewer #2:** The article titled “Species-specific emergence of highly pathogenic avian influenza virus is driven by intra-host selection differences between chickens and ducks” is a comprehensive experimental study aimed at analyzing the species-specific differences in the selection of different pathotype of H7 avian influenza virus using a mixture of the two at a 1:62 dilution in chickens and ducks. The authors were able to trace LP- and HP-AIV pathotypes by engineering specific tags on the HA. The manuscript is well written, and the research rationale is sound. There are only a few details that need clarification.

**Reviewer #3:**

Strengths:

- The paper examines an important topic: the selection of highly pathogenic influenza viruses within natural hosts. This is particularly relevant as HPAIVs currently pose a tangible threat to animals and humans.
- The hypothesis tested is clear and the results support it.
- The system used is of utmost biological relevance (the results presented are from in vivo experiments using natural hosts).
- Originality: I am not an expert on avian influenza and thus I am not sure if the results presented are original, or if they constitute incremental knowledge. Based on the discussion, it seems that the latter is the case.

We thank the reviewer for their positive remarks. With regard to the originality of our work, we would like to emphasize that this is the first study in which the intrahost selection of HPAIVs is compared between model species for poultry and wild aquatic birds and concomitantly in which a pair of viruses representative of a natural LPAIV to HPAIV conversion is utilized. In previously published work on intrahost competition, which is thoroughly discussed in the Discussion Section, either only the selection in poultry was investigated (PMID:30514922 and PMID: 31969434) or a pair of viruses based on a Goose/Guangdong-lineage H5N8 HPAIV was utilized, i.e., by Bessière et al. (PMID:34613804). Both the HPAIV and LPAIV in this latter pair of H5N8 viruses did not result in disease and replication phenotypes in chickens nor ducks that reflect those of natural LPAIVs or non-Goose/Guangdong HPAIVs. Therefore, the conclusions drawn in the study by Bessière et al. on selection differences between chickens and ducks are not applicable to natural LPAIV to HPAIV conversions. To emphasize the novelty of this work, one sentence has been added to the Discussion (lines 489-490).

Weaknesses:

- The main weakness is that the host response to infection by LP or HP or coinfections, was not studied. This is a shame because i) it would likely provide very important information about the processes that drive the observed differences in tropism, shedding and selection; and ii) because the authors have the materials and expertise to tackle this issue easily (see major comments below).

We thank the reviewer for their critique. We have extensively commented on the proposed research and corresponding concerns in Part II ‘Major issues’.

**Part II – Major Issues: Key Experiments Required for Acceptance**

*Please use this section to detail the key new experiments or modifications of existing experiments that should be absolutely required to validate study conclusions.*

*Generally, there should be no more than 3 such required experiments or major modifications for a "Major Revision" recommendation. If more than 3 experiments are necessary to validate the study conclusions, then you are encouraged to recommend "Reject".*

**Reviewer #1: Major concerns:**

1. Even in the mono-inoculated H7N7-HP ducks, the authors barely detect any infectious virus by TCID50 assay. Is it possible that the infectious dose of HP needed for productive infection in ducks is higher that the dose needed for chickens? These results would be convincing if the authors could perform H7N7-HP (10^6) infections in ducks at a higher dose and establish productive infections.

We concur that the amount shedding of infectious HPAIV in the mono-inoculated ducks is not high. However, shedding of infectious virus was present in all ducks on 3-6 dpi, which suggests productive infection as this timing corresponds to the detection of newly produced virus rather than leftover inoculum. Additionally, infectious HPAIV was robustly detected in tissues at 3 dpi (Fig. 3D), also suggesting productive virus replication. Furthermore, the use of a higher inoculation dose, such as 10^6, would defeat the purpose of the experiment to investigate HPAIV intrahost selection. The inoculation ratio was purposefully set at 1:100 HPAIV:LPAIV as this mimics the first steps of HPAIV emergence, with competition with a larger population of replicating LPAIVs. Matching the inoculation doses to a ratio of 1:1 would not represent the natural sequence of events during HPAIV emergence. Rather, we think that the low amount of productive HPAIV replication detected in the mono-inoculated ducks is a result of restricted virus replication of HPAIV ducks, which has been described in the literature as indicated in the introduction and was the basis of our hypothesis, eventually leading to the absence of HPAIV intrahost selection. Taken together, we do not think that experiments with a HPAIV 10^6 inoculation dose are essential to be able to draw the conclusions presented in this manuscript.

2. While I agree with the overall conclusions, additional co-inoculation studies in chickens and ducks will strengthen their findings. These experiments were done once with an n=3 for each time point. On Day 7, only 1 out of 3 chickens have high HPAIV titers. Similarly, on day 3, 1 out of 3 chickens have detectable LPAIV. As there is a lot of variation between birds, additional experiments will help rule out any stochastic effects.

The study design was purposefully set up to focus on qualitative rather than quantitative assessments, as usually done for experiments with large animals due to ethical considerations. We performed detailed and thorough investigations on each animal sample, applying orthologous methods to determine virus replication, for example the determination of viral load by RT-qPCR, end-point titration assay, and plaque assay and additionally for tissues by IHC and ISH. To complement the data from viral shedding, virus presence in the environment was investigated. The combination of this number of different analyses is unique in the literature regarding avian influenza virus infections in birds and allowed a strong qualitative assessment of the experiments. We concur that there is a lot of variation between the birds, but, as mentioned in the Discussion (line 417), some degree of stochasticity is expected during intrahost virus selection. Despite this, patterns of HPAIV and LPAIV selection were clear in both chickens and ducks. HPAIV was selected in four out of six chickens, based on the development of canonical HPAI signs, high HPAIV infectious virus shedding and detection of HPAIV infectious virus and RNA in the drinking water. In contrast, LPAIV was favored in ducks in the long-term, as exemplified by the absence of infectious HPAIV shedding in any of the ducks at any point in time and the absence of HPAIV infectious virus and RNA detection in the drinking water. Furthermore, we do not think that the doubling of the number of animals used in this study can be defended to an ethical committee as this would only strengthen the findings rather than change the overall conclusions drawn.

3. The organization of supplementary figures in the manuscript needs to be changed. In the current manuscript, body weight, viral RNA, viral titers are organized in separate supplementary figures. While it is easy to compare these parameters across different viruses, it is really hard to follow the results section when panels from different supplementary figures are recalled.

We agree with the reviewer that the manner in which the supplementary figures are presented is not ideal. However, we have attempted rearranging the data in the way the reviewer suggests, and the resulting supplementary figures did not aid the readability of the manuscript as it increased the number of supplementary figures substantially. We think that the grouping of the data in the current format is the most optimal way of displaying the high amount of gathered data.

**Reviewer #2:**

1) Could the author expand on the rationale behind the 1:62 mixture (prior literature)?

The inoculation doses aimed for in the experiments were 10^4 EID50 HPAIV and 10^6 EID50 LPAIV, which upon back-titration of the co-inoculation inocula in embryonated eggs lead to a final ratio of 1:62 rather than the intended 1:100. The rationale behind this ratio is as follows: as this study aimed to compare HPAIV selection between bird species, it was imperative to use a ratio in which HPAIV can result in productive infection in both species upon mono-inoculation. It is ethically not allowed to perform animal experiments in the Netherlands with the sole purpose to determine BID50 values, which would have allowed for the determination of the minimal infective dose of the HPAIV. Instead, 10^4 EID50 was chosen as 10^4 EID50 resulted in the productive infection of all ducks upon inoculation of a chicken-isolated HPAIV and often results in productive infection in ducks in literature (PMID:31068421, PMID: 28481948). To enhance clarity in the manuscript, the 1:62 ratio was removed from the abstract and introduction, as it is a ratio determined by back-titration rather than the intended 1:100 ratio, and the intended doses are indicated instead (line 100-101 and 227-228). Furthermore, an explanation for the 1:100 ratio has been added (line 100-101).

2) In both mono- and co-infections, the authors observed widespread and elevated HPAI virus tissue distribution in all infected birds compared to the limited tissue distribution to only gastrointestinal tract of ducks with LPAIV (compatible with previous literature). However, when looking at virus shedding values, it seems that LPAIV is shed OP and CL swabs to higher extent than HPAIV in both chickens and ducks which is counter intuitive. Could the authors comment on the opposite results obtained from tissues compared to swabs?

To comment on the reviewer’s first concern on the seemingly higher shedding of LPAIV than HPAIV, it is important to note that the inoculation doses differed by a 100-fold between the LPAIV and HPAIV in the mono-inoculations. Therefore, the initial shedding levels of LPAIV, which was actually only shed via the OP route and not via the CL route in chickens, are expected to be higher than those of HPAIV. However, in chickens, OP shedding levels of HPAIV increased to comparable levels around the time that the tissues were harvested (3 dpi) and, as expected, the HPAIV was detected at higher infectious levels and in more tissues than the LPAIV, which is in accordance with literature as the reviewer notes.

To comment on the reviewer’s second concern regarding the opposite results obtained from duck swabs and tissues, we concur that there is a difference in detection of infectious HPAIV in tissues and swabs especially of co-inoculated ducks. Therefore, we had devoted a paragraph in the Discussion (lines 474-488) to this difference. The reasons behind these discrepancies are not entirely clear, but might have to do with virus stability or immune responses.

3) Beside the MBCS, did the H7 virus genome contained any other virulence marker or markers of mammalian adaptation within other genes?

Markers for virulence in birds are not as well mapped as those for virulence in mammals. To our knowledge, HPAIV A/chicken/Netherlands/1/03 does not contain any known markers of adaptation to poultry or mammals.

4) Fig 7 recapitulates the tissue distribution of virus antigen as represented by nucleoprotein and HAtag. In subpanel F (C20 co-inoculation), the figure shows positivity for nucleoprotein and not the HA-tag, but in the summary panel H, both HA-tags and FLAG-tag are marked positive. Could the authors demonstrate or comment on the interpretation since as it is displayed seems contradictory?

The data shown in Fig. 7 panel HA is correct. Indeed, Fig. 7 panel F only shows a picture of the LPAIV-positive area found in the bursa, but a picture of the HPAIV-positive area was shown in Supplementary Fig. 12, which was mentioned in the main text. To avoid confusion, Fig. S12 has now been added to panel F of Fig. 7.

5) The authors analyze the differential replication of tagged and untagged HP and LP viruses in vitro and in chickens. Is there a reason why the authors did not analyze mono-untagged H7 HPAIV replication in ducks? Differential replication in vivo would be important to further support the authors conclusions on competitive advantage of tagged LP and HP in ducks.

We purposely opted to compare in vivo replication kinetics of tagged with untagged viruses in one species only, i.e., the species which is the main host of the corresponding virus, to reduce the number of animals involved in the study. Consequently, the tagged and untagged LPAIVs were compared in their original reservoir host consisting of ducks (Fig. S3) and the tagged and untagged HPAIVs were compared in chickens (Fig. S5). In the final competition experiments, only tagged LPAIV and HPAIV were compared.

**Reviewer #3:** The following experiments are not absolutely required. They would make this study much more comprehensive and would provide insights that the study as it stands does not provide.

The authors should examine the host response to infection to the viruses (in single and coinfections) as this will likely provide important information about the differences observed in tropism, shedding and selection. This could be done by using the FFPE tissues that had been collected, and use IHC or IF to quantify markers of (for example) IFN response, inflammation, or any other response that they think could contribute to the observed differences. If antibodies are not available, they could perform RNAscope. The data presented in the manuscript shows that they are very capable of doing this.

Alternatively, or complementarily, they could do transcriptomics with the RNA they obtained from tissues. This might not be as straightforward as it would imply doing experiments (RNAseq) and analyses (bioinformatics) that had not been included in the original manuscript.

We thank the reviewer for their suggestions, which are not deemed necessary to validate the study conclusions to their opinion. The study design of the experiments described in this manuscript was chosen to allow testing the hypothesis whether HPAIV intrahost selection differs between bird species. This hypothesis arose from the plethora of data presented in the literature about the well-described differences in HPAIV infection kinetics, tropism, and host responses between species of terrestrial poultry and wild aquatic birds. We therefore did not set out to characterize host responses in this study design as they were seen as a starting point of our research rather than an endpoint. This study therefore stands on its own and will subsequently allow further in depth follow up of the role of host responses on intrahost virus selection. However, in order to properly compare host responses between the species, another study design than the one used in this study would be preferred. For example, the 3 dpi time point allows the investigation of HPAIV selection, but is too late for the investigation of host responses leading to HPAIV selection, especially in chickens. Furthermore, to allow direct comparison of host responses between viruses, the inoculation doses would preferably be the same and not consist of the 1:100 ratio as aimed for in this study (10^4 and 10^6 EID50). We would indeed be very interested to relate the host responses of chickens and ducks to HPAIV selection in follow up studies, especially in a single cell context using RNAscope. However, we would completely change the design of the study with this aim in mind.

**Part III – Minor Issues: Editorial and Data Presentation Modifications**

*Please use this section for editorial suggestions as well as relatively minor modifications of existing data that would enhance clarity.*

**Reviewer #1: Minor concerns.**

1. Fig S2C – please include legends to indicate that they are co-inoculated animals.

The legend has been added accordingly.

2. LN 178 - it is not clear what chicken C15 refers to in Fig 3A and C.

The chicken identifier has been removed as it is clear that the one chicken whose tissues show high titers is indicated by the triangle symbol in Fig 3 (line 185).

3. In Fig 4 – the quantification of FLAGtag and NP would be a better representation of LP strain than NP+ and HAtag-, as some of the viruses may be defective in expression of viral hemagglutinin protein with HAtag.

We agree with the comment of the reviewer that FLAGtag and NP co-stainings would have been a better approach. Unfortunately, we noticed aspecific background staining by anti-FLAG antibodies in infected cells. This precluded performing any FLAGtag/NP co-stainings. This failure to reliably detect FLAGtag is mentioned in both the Results section (line 236) and Materials and Methods section (lines 670-671 and 694-695). Nevertheless, Fig. 4 shows data generated with plaque assays in which individual plaques were either HAtag-negative or HAtag-positive upon HAtag and NP immunostaining. If, as the reviewer suggests, a single virus particle in the sample would not contain viral hemagglutinin, i.e., be defective, a plaque would not have been generated at all and would therefore also not be falsely identified as HAtag-negative and NP-positive. Furthermore, it can be assumed that the cells within a plaque are not in the same stage of the virus replication cycle, therefore a subset of cells within each plaque will always be in the stage in which HA is produced.

4. In LN 383-385 - it is not clear what the authors refer to by “co-sampled airsac”. Does this mean that there was tissue contamination during harvest?

The assumption from the reviewer is correct. Because of the omnipresence of air sacs in the body of the animals, it cannot be excluded that remnants of air sacs remained attached to some other tissues despite us being extra cautious during dissections. The possibility for cross-contamination by air sacs during dissection has been explicitly stated in lines 401 and 482-483.

5. In LN394 - HPAIV is naturally selected in 4/6 chickens. The term “naturally” can be misconstrued and should be replaced with HPAIV had a selection advantage over LPAIV or something similar.

The term ‘naturally’ has been removed from the sentence and replaced with ‘intrahost’ (line 345), which is the term used to describe the type of selection investigated in this study.

**Reviewer #2:**

6) Were the immunostainings interpreted by a pathologist blind to the treatment group? If so, please specify.

The immunostainings were not interpreted in a blinded fashion by a pathologist. We instead opted for an unambiguous 3-level scoring system which was performed by one virologist for standardization. Furthermore, we refrained from any overarching quantitative analyses.

7) For clarity, I would suggest the authors add “H7” in the title.

Title was adjusted accordingly.

**Reviewer #3:**

**Disclosure**: I am not an expert on avian influenza. I do have expertise on mammalian influenza viruses.

In this manuscript, de Bruin et al. posed a clear and testable hypothesis: that intrahost selection of HPAIVs is favoured in poultry and not in ducks. They also hypothesize that this is due to differences between bird species in virus tropism, ability to disseminate systemically and virus replication. To test their hypothesis, the authors performed a pathogenesis study to examine the process of selection of highly pathogenic avian influenza virus (HPAIV) in vivo, using a compare/contrast approach based on experimental infections and coinfections of chicken and ducks. Specifically, they used two isogenic viruses generated by reverse genetics: the wild-type H7N7 HPAIV virus (A/chicken/Netherlands/1/03) and a low pathogenic version in which the HA polybasic region of the virus was replaced by a low-pathogenic sequence. The viruses used were further modified by the addition of epitope tags to allow discrimination between them.

Chickens and ducks were subject to experimental inoculations with HPAIV and LPAIV viruses in single and coinfections. Inoculated animals were monitored for clinical signs; virus shedding was measured by qPCR and titration of infectious virus; and distribution of virus antigens was examined the respiratory and the gastrointestinal tract, as well as other tissues.

The study is very well designed and executed. The in vivo nature of the study, using chickens and ducks, which are natural hosts of avian influenza viruses, gives this study a very high biological relevance. I think that, overall, the results support their hypothesis. The interindividual variation observed in shedding and virus detection in tissues is expected, so I do not have a problem with it.

We thank the reviewer for their positive critique and we share their opinion that interindividual variation is expected in experiments of this nature.

I found the manuscript difficult to follow, but this might be simply because there is so much data presented. However, some points could have been clearer. For example, the sentence that describes the process of HPAIV emergence (genesis, selection, and transmission, line 482) should be in the introduction as it would help setting up the focus of the study.

The three steps of HPAIV emergence have been added to the introduction (lines 88-93).

My main criticism about the study is that the authors did not go a little bit deeper to identify the main processes involved in differential selection of HPAIV and LPAIV in chicken and ducks. Since they have RNA from tissues, as well as FFPE sections, they could have explored differences in the host response to infection and coinfection that might explain the observed results. For example, are there differences in the IFN response in tissues? Are there differences in inflammatory responses and/or lesions that could explain the observed differences in virus antigen distribution? Transcriptomics or immunostaining approaches could provide important information

The issue of the lack of investigation into host responses has been addressed previously in the response to Reviewer 3 in Part II.

Another main comment is about novelty. The authors mention in their discussion that their results are mainly consistent with previous studies. They also mention some differences with other studies but discuss potential differences appropriately. As I am not an avian influenza expert, I cannot say if the results presented are of enough novelty for PLOS PATH.

The issue of novelty has been addressed previously in the response to Reviewer 3 in Part I. To emphasize the novelty of this work, an extra sentence has been added to the Discussion section (lines 489-490).

*Minor comments:*

- It is a shame that the FLAG-tag signal was non-specific. As a result, the identification of the FLAG-tagged virus was inferred rather than directly detected.

We completely agree with Reviewer 3 that a FLAGtag staining would be preferable, but as explained before in an answer to comment 3 of Reviewer 1, this was not feasible.

- It would be good to state it in the paper the rationale for the 1:62 ratio of HP/LP viruses in coinfections.

This was done accordingly (see answer to comparable comment of Reviewer 2).

- What was the criterion for tissue sample collection? Did you collect tissues from areas that exhibited macroscopic lesions? This is important for organs such as the intestines that are quite large.

Sample collection was standardized as much as possible and not based on the presence of macroscopic lesions. Instead, the pieces of tissue were collected from the same location across all the animals. Due to its length, the small intestine was the only tissue in which the exact part taken for analysis might not have been the same in all animals.

- Regarding qPCRs, was viral genomic RNA quantified? If yes, it would be simpler to present the results as genome copies/gram of tissue. I don't know how to interpret the RNA quantities as presented --> (40-Ct) normalized/gram.

The one-step RT-qPCR used in this study do not discriminate between negative-sense and positive-sense RNA and therefore both genomic vRNA, present in viral particles and infected cells, and cRNA/mRNA, present in infected cells, are amplified when homogenized tissues are used as starting material. The unit ‘40-Ct/gram’ covers all types of RNA amplified during the RT-qPCR, whereas ‘genome copies/gram’ does not.

- Have the viruses been sequenced when collected from the animals? Were their sequences the same as what was inoculated? Is it possible that the interindividual variations observed in virus shedding and virus distribution were due to changes in the virus at the consensus level?

Only a subset of 3 dpi and 7 dpi tissues from chickens and ducks mono-inoculated with tagged viruses was tested for the presence of the tag in HA. No other sequencing analyses were performed. Unfortunately, it would not be possible to correlate the interindividual variation of the co-inoculation experiments to changes on the vRNA sequence level, because the two viruses used in the experiments are isogenic except for the cleavage site and the epitope tag. We would therefore not be able to attribute a certain genetic change to one of the virus variants. A different approach would have been necessary to draw conclusions on that level, for example by adding single nucleotide polymorphisms to the entire genome of the two viruses to be able to differentiate them by long-read next generation sequencing. Furthermore, as single substitutions in specific avian influenza strains are not as well characterized in certain human influenza strains, characterization of the detected mutations would require further animal experimentation to be able to draw any strong conclusions on their influence in virus intrahost selection.

- The graphs should have the same scale in the Y axis for all the figures.

We are of the opinion that our current approach, fitting the largest value present within the graph with the Y axis, represents the data well and that changing all the Y axes to the same value will reduce the ease at which the data can be interpreted. Importantly, the Y axes of graphs from different panels in the same figures and of graphs from Fig 4 and Fig 5 (describing data collected from the co-inoculated animals) are the same.

- In figure 6, I do not understand the difference between a score of 0 and "not present". Please clarify.

Not present means that the tissue was not present and thus the analysis was not performed. A score of ‘0’ refers to the absence of NP staining in the analyzed tissue. For clarity, the abbreviation will be changed to ‘not performed’ (line 340).

*Editorial changes and clarifications suggested:*

- Replace sacrificed with euthanised throughout the text of the paper (sacrifice has religious implications).

The wording has been adjusted accordingly, both in the manuscript and in the supplementary materials.

- For each result it would be good include a final sentence summarising the overall result. This was done for some results but not for others. Given the complexity of the experiments and data presented I think this is very useful even if it may sound repetitive.

Summarizing sentences were added accordingly (Lines 154, 158, 298-299, 331-333, 371-373, 401-402).

- Line 375: "In co-inoculated ducks, NP expression was detected in a few cells in the air sacs and colon (Fig 6B), which constituted of too few cells to robustly perform the HAtag immunostaining." However, in Figure S12 you show a single cell that is positive for NP and on the next serial section you show a single cell that is positive for HAtag. There seems to be no reason not do the HAtag staining.

The reasoning behind the choice to just perform co-stainings on the tissues scored as ‘2’ in NP-staining constituted of the fact that the distinction between HPAIV and LPAIV was based on the co-detection of NP and HAtag expression and the size of the infected area. Tissues scored ‘2’ contained enough cells in a certain area which spanned multiple sequential FFPE slides to be able to confidently state whether the sequential slide was HAtag negative or positive. However, the chances were very high that when only one cell is NP-positive on the first slide, the sequential slide would not contain that cell anymore and would falsely be designated as HAtag-negative and therefore H7N7-LP_FLAGtag_ positive. Therefore, we cannot confidently designate which virus is replicating in the tissues scored ‘1’. The reason why Fig S12 was included in the manuscript is because the tissue was originally scored ‘2’ due to the large infected area shown in Fig. 7 panel F. The NP- and HAtag-positive cell shown in Fig S12 was detected by chance. To increase clarity, Fig S12 has now been incorporated in panel F of Fig. 7.

- Line 436: "Here, the replication of H7N7-HPHAtag in air sac epithelial cells of co-inoculated chickens at 3 dpi was striking. " Please explain why it was striking.

An explanation was added to the text (lines 454-455): “as it was present in all three co-inoculated chickens whereas it was absent in mono-inoculated chickens”.

- Line 456: "in tissues outside the respiratory and digestive tracts in ducks suggesting". Is this referring to coinfected ducks? Or monoinfected? Please clarify in the text.

HPAIV was detected outside the RT and GIT in both mono- and co-inoculated ducks and LPAIV was detected outside the RT and GIT in mainly mono-inoculated ducks. Therefore the addition “mono- and/or co-inoculated ducks” was added to the text (line 475).

- Line 460: "Here, we have found no convincing evidence of active LPAIV or HPAIV replication, either by detection of viral antigen or genomic viral RNA, outside of the respiratory and digestive tracts of ducks." I think Figures 3 and 5 contradict this statement. Figure 3 shows RNA of both HP and LP viruses in adrenal gland, bursa, and kidney. If I am wrong, please clarify what is meant by "convincing" evidence.

Direct evidence of active replication was defined as the detection of virus-infected cells in the FFPE samples, by IHC or ISH, rather than only the detection of RNA in homogenized tissues. This specification has been added to the main text for clarity (lines 479-480). Virus RNA-positivity of homogenized samples could be caused by low levels of cross-contamination or abortive infections and is therefore not always the result of active replication.
