# Supplementary material for: Species-specific emergence of H7 highly pathogenic avian influenza virus is driven by intrahost selection differences between chickens and ducks
Source: PLoS Pathog. 2024 Feb 26;20(2):e1011942. doi: 10.1371/journal.ppat.1011942 (PMC10919841; doi:10.1371/journal.ppat.1011942)
Supplement: Attachment — Submitted filename: response to reviewers.docx [file ppat.1011942.s016.docx]

We thank the reviewers and the editorial board for their positive assessment of the revised manuscript. Regarding the minor revisions, please find the reviewers’ comments reproduced below, followed up by each of our answers in a green font. Line numbers refer to the ‘Marked-up Manuscript’ file.

**Part I – Summary**

Reviewer #1: I am fine with the authors responses. In lieu of performing additional animal experiments, the authors should consider including a section about the limitations of the study (animal numbers, differences in HPAIV replication in duck vs chickens etc) and indicating that these experiments are designed to be qualitative.

In response to the suggestion of Reviewer 1, we have emphasized in multiple sections that the manuscript describes an in-depth and qualitative assessment of HPAIV intrahost competition. This includes the introduction (line 96) and an additional section early in the discussion (lines 405-409).

Reviewer #3: This is a submission of a revised manuscript. The authors provided a point by point response to the reviewer's comments. My review of the revised version is described below (see major and minor comments).

**Part II – Major Issues: Key Experiments Required for Acceptance**

Reviewer #1: none

Reviewer #3: The authors have justified why additional *optional* experiments suggested by me are not needed. As they were -in my view- optional I am OK with their decision. Besides, the lack of such additional data does not detract from their findings.
I am in two minds about the request by another reviewer about including more animals: on one hand I see that inter individual variation is expected and for this reason numbers matter (i.e. you need larger numbers to observe clear differences). On the other hand I understand issues about logistics and ethics when using animal species that are not as common as mice.

We have taken note of the optional experiments that reviewer 3 suggested in the initial round of peer review and we have the ambition to implement these in our follow-up studies. The differences in virus selection between the species were large, despite the interindividual variation, and thus we believe that our animal numbers are sufficient. We added one sentence in the discussion (line 518-519) to emphasize this point.

**Part III – Minor Issues: Editorial and Data Presentation Modifications**

Reviewer #1: none

Reviewer #3: The authors addressed most of the minor issues raised. They also provided proper justification for the minor issues they did not address.
